# Association between Age and Severity to Leptospirosis in Children

**DOI:** 10.1371/journal.pntd.0002436

**Published:** 2013-09-26

**Authors:** Gilles Guerrier, Pauline Hie, Ann-Claire Gourinat, Emilie Huguon, Yann Polfrit, Cyrille Goarant, Eric D'Ortenzio, Isabelle Missotte

**Affiliations:** 1 Intensive Care Unit, Centre Hospitalier Territorial, Noumea, New Caledonia; 2 Paediatrics, Centre Hospitalier Territorial, Noumea, New Caledonia; 3 Laboratory of Virology, Institut Pasteur in New Caledonia, Noumea, New Caledonia; 4 Laboratory of Microbiology Research Unit, Institut Pasteur in New Caledonia, Noumea, New Caledonia; 5 Infectious Diseases Epidemiology Unit, Institut Pasteur in New Caledonia, Noumea, New Caledonia; Universidad Peruana Cayetano Heredia, Peru

## Abstract

**Background:**

In endemic areas, leptospirosis is more common and more severe in adults compared with children. Reasons to explain this discrepancy remain unclear and limited data focusing on adolescents are available. The objective of the study was to describe disease spectrum and outcome differences in children and adolescents admitted for leptospirosis in a large at-risk population.

**Methods:**

Clinical and laboratory data were obtained on hospitalized cases in New Caledonia from 2006 to 2012.

**Results:**

Data of 60 patients <18 years of age (25 children under 14 and 35 adolescents aged 14 to 17) with confirmed leptospirosis were analyzed. Compared with children, adolescents presented more often with classic features of Weil disease (p = 0.02), combining hepatic and renal involvement with or without pulmonary participation. Jarisch-Herxheimer reactions were observed more often among adolescents (p<0.01). The overall case fatality rate was low (1 adolescent versus 0 children).

**Conclusion:**

Severe leptospirosis in adolescents may be more likely to show adults' characteristics compared with children. Further studies are required to explore age-dependant host factors, including puberty-related physiological changes.

## Author Summary

Leptospirosis is endemic in tropical areas and seems to affect adults more often and more severely than children. Factors responsible for such differences have not been clearly established. However, host-related factors are believed to play a role in the development of severe leptospirosis. The study aimed to describe disease spectrum and outcome differences in confirmed cases in children and adolescents in New Caledonia. One major finding is the milder presentation of children compared with adolescents. Clinical and biological characteristics in adolescents are similar to adults, including occurrence of Jarisch-Herxheimer reactions. Further studies are required to explore age-dependant host factors, including puberty-related physiological changes.

## Introduction

Leptospirosis is an important zoonosis of worldwide distribution caused by pathogenic spirochaetes of the genus Leptospira. Humans usually become infected through contact with water or soil contaminated by the urine of mammalian reservoirs such as rodents, dogs, cattle and pigs [1].

Infection most commonly results in asymptomatic or self-resolving illness both in adults and children [2]. In severe cases requiring hospitalization, the disease is however potentially fatal classically presenting with jaundice and renal dysfunction (Weil disease) with or without pulmonary hemorrhagic manifestations. According to estimates from the World Health Organization, more than 500,000 severe cases occur every year worldwide, mostly in tropical and sub-tropical regions.

Along with experienced clinicians' beliefs, several studies suggest leptospirosis to produce more severe presentation in adults compared with children [3], [4], [5], [6], [7]. Pathogen as well as host-related factors are believed to play a role in the development of severe leptospirosis in adults [1], [8]. However, factors responsible for the milder presentation among children remain unclear. There is limited information available about symptomatic leptospirosis in the under 18 age group in the Pacific region.

New Caledonia is an overseas French-administered territory located in the South Pacific and located 1500 kilometres East of Australia. According to the 2009 census, the population is 245 580 inhabitants with a mean increase of 1.7% per year since 1996 [9]. The population has good access to the health care system of European standards. Climate in New Caledonia is marked by a cool and dry season (from June to September) and a warm and wet one (from December to March). Leptospirosis is endemic to New Caledonia, and is a leading cause of hospital admission during the rainy season [10], [11]. From 2006 to 2009, the average annual incidence was 45 cases per 100,000 inhabitants but reached 150 per 100,000 inhabitants during the rainiest months.

The objective of the study was to describe disease spectrum and outcome differences in children and adolescents admitted for laboratory-confirmed leptospirosis in a large at-risk population. We hypothesized that adolescents were more likely to present with all the classic features of Weil disease compared with younger children.

## Methods

### Study design

We carried out an observational retrospective study among patients aged under 18 years old with a biologically confirmed leptospirosis admitted between January 2006 and December 2012 in either of two public hospitals (Centre Hospitalier Territorial, Noumea and Centre Hospitalier du Nord, Koumac). Demographic, epidemiologic, clinical, and laboratory information were recorded. Outpatients testing results for leptospirosis or adults over 18 were not included in the analysis. Leptospirosis was defined by a compatible clinical syndrome (any combination of fever, chills, myalgia, jaundice, conjunctival suffusion, renal failure, hemorrhage, or pulmonary failure) and laboratory confirmation with one or more of the following features: 1) positive results for a microscopic agglutination test (using a panel of 11 serovars) and one acute-phase serum sample titer >1∶800, 2) seroconversion between times of testing acute-phase and convalescent phase serum samples, 3) a four-fold increase in titers between two examinations, or 4) a positive PCR. Biological tests performed at the Institut Pasteur in New Caledonia (IPNC) have been described in a previous study [8].

Patients were stratified by age group: 0–13 years of age (children) and >13 years (adolescents). Oliguria was defined as urine output <0.5 mL/kg/hour and pulmonary involvement was defined as dyspnea, rales, and/or chest radiographic abnormalities. Laboratory results refer to samples collected at the time of admission. Reference values were serum creatinine  = 30–88 µg/dL, total bilirubin = 0.8–1.2 mg/dL, and platelet counts = 150 000–400 000/mm3. The time (in days) between onset of symptoms and hospitalization was compared between groups. Occurrence of Jarisch-Herxheimer reaction (JHR) was also noted when reported. JHR was defined as the combination of sudden onset of shivering or rigors, with rise in temperature, with or without a fall or a rise in blood pressure, increase of respiratory rate occurring after administration of the first dose of antibiotics.

### Procedure and data collection

For each study participant, a standardized form was retrospectively completed. Clinical manifestations and medical history were collected as mentioned in medical records. Demographic data and laboratory results were extracted from electronic records. Both methods (MAT and PCR product sequence polymorphism) were used to identify the serogroup or the putative serogroup of the infecting strain.

### Ethics statement

The study was approved by the Institutional Review Board of Centre Hospitalier Territorial. Informed consents were not obtained from the patients as this was a retrospective study. All data were anonymized.

### Statistical analysis

STATA (Stat Corp., College Station, TX) was used for analysis. Quantitative and qualitative variables were compared by using paired Student's t-tests and chi-square tests, respectively. When the frequency of events was <5 or values did not follow normal distributions, Fisher's Exact and Mann-Whitney tests were used.

## Results

A total of 128 patients with leptospirosis were diagnosed during the study period. Sixty eight of these cases were excluded from further analysis because they were not hospitalized in one of the two participating centres (n = 50) or records could not be traced (n = 16).

Of the 60 patients included in the study, 25 (42%) were children and 35 (58%) were adolescents. The majority of study subjects were boys (79% in children vs 77% in adolescents, p = 0.6) with a mean age of 9 [IQR 6–11] years for children and a mean age of 16 [IQR 14–17] for adolescents. Subjects were mostly Melanesian (83%) living in tribes in rural areas (85%) (Table 1).

**Table 1:** Demographic characteristics and medical histories of 60 paediatric leptospirosis in New Caledonia, 2006.

Characteristic	Children n (%)	Adolescents n (%)	p-value
Gender			
Male	18 (72)	27 (77)	0.6
Female	7 (28)	8 (23)	
Age (Mean [25–75 IQR])	9 [6–11]	16 [14–17]	
Ethnic group			
Melanesian	19 (76)	31 (88)	0.3
Other community	6 (24)	4 (12)	

The frequency of symptoms and complications are presented in Table 2 and biological parameters are presented in Table 3. Fever and jaundice were more frequent among adolescents whereas the incidence of conjunctival suffusion, myalgia, abdominal pain, headache and oliguria were not significantly different between groups. Mean total serum bilirubin and serum creatinine levels were higher in adolescent than paediatric groups (41 versus 7; p<0.001 and 108 versus 59; p<0.001, respectively). Platelet count was lower in adolescents (148 000 [95%CI 124 000–173 000]) than in children (202 000 [95%CI 157 000–248 000]) (p = 0.02). Compared with children, adolescents presented more often with classic features of Weil disease (p = 0.02), combining hepatic and renal involvement with or without pulmonary participation. The groups showed no differences with respect to pulmonary involvement.

**Table 2:** Clinical symptoms in 60 leptospirosis children in New Caledonia, 2006–2012.

Clinical presentation at admission	Children n (%)	Adolescents n (%)	p-value
Fever (>38°) at admission	10 (40)	23 (66)	0.04
Jaundice	1 (4)	10 (28)	0.03
Headache	16 (64)	27 (77)	0.3
Abdominal pain	11 (44)	22 (63)	0.1
Myalgia	18 (72)	25 (71)	0.9
Conjonctival suffusion	4 (16)	9 (26)	0.4
Oliguria	2 (8)	7 (20)	0.2
Pulmonary involvement (cough, dyspnea, auscultation abnormalities)	7 (28)	11 (31)	0.6
Weil disease	0 (0)	7 (20)	0.02
**Complications**			
Alveolar hemorrhage	1 (4)	5 (14)	0.2
Myocarditis	0 (0)	1 (3)	0.5
Case-fatality rate	0 (0)	1 (3)	0.5

**Table 3:** Initial laboratory findings among 60 leptospirosis children in New Caledonia, 2006–2012.

Parameter*	Children n = 25	Adolescents n = 35	p-value
Bilirubine (mg/dL)	7 [5–12]	41 [34–55]	<0.001
Creatinine (µM/dL)	59 [62–65]	108 [85–132]	<0.001
Platelet count (G/L)	202 [157–248]	148 [124–173]	0.02

* mean [95%CI].

Exposition factors for leptospirosis transmission did not differ between the groups; most patients in both groups had self-reported direct contact with water (swimming in rivers or canals, wading through water) (87% in children and 81% in adolescents), and direct or indirect contact with mammalian carriers (25% in children and 28% in adolescents).

The median time between onset of symptoms and initiation of antibiotics was 2 days for both groups. Antibiotics were administered intravenously for 5–7 days and included ampicillin (100 mg/kg of body weight/day) in 43 patients (18 children and 25 adolescents) and cefotaxime (100 mg/kg of body weight/day) in 17 patients (7 children and 10 adolescents).Jarisch-Herxheimer reactions were observed more often among adolescents (p<0.01) (Table 4). The overall case fatality rate was 1.6% (1 adolescent versus 0 children). The single patient with fatal outcome required dialysis for acute renal failure, mechanical ventilation for alveolar hemorrhage and vasoactive drug for shock. Four other adolescents required vasoactive drugs only. No other patients required dialysis or mechanical ventilation.

**Table 4:** Characteristics of infection and effects of antibiotics among 60 leptospirosis children in New Caledonia, 2006–2012.

	Children n (%)	Adolescents n (%)	p-value
**Infection serogroup**			
L. interrogans serogroup Icterohaemorrhagiae	7 (64)	8 (53)	0.7
Others	4 (36)	15 (47)	
**Duration of symptoms before admission to hospital (days)** *	2 (1–3)	2 (1–3)	0.2
**Jarsich-Herxheimer reaction**	1 (4)	12 (34)	0.005

* mean [95%CI].

Among the 26 cases for whom the serogroup was identified, 15 (58%) were Icterohaemorrhagiae (7/11 in children, 8/15 in adolescents). Other detected serogroups included Australis (n = 4), Pyrogenes (n = 4), Canicola (n = 2), and Panama (n = 1) (Table 3). For the 34 remaining cases, identification of the serogroup was not possible. At the time of admission, 8 patients had acute co-infections (5 children and 3 adolescents) with viral diseases (rotavirus n = 2; viral respiratory syncitial n = 1), or bacterial diseases (bacteraemia with Serratia n = 1; urinary tract infection n = 2, pyodermititis n = 1, tuberculosis n  = 1). All patients diagnosed with coinfections were leptospirosis confirmed cases by PCR.

## Discussion

This retrospective study allowed us to identify an age-dependant association with severity of leptospirosis. Similar observations in children have been reported in other settings [3], [12]. The frequency of several classic severe disease manifestations were significantly lower among small children in this study compared with adolescents. Our study found that a substantial proportion of hospitalized children with leptospirosis had fewer of the classic features of Weil disease than adolescents. Although severe disease caused by leptospirosis may occur in the paediatric age group, clinical and biological presentation in adolescents overlaps with the spectrum seen in adults. Since most studies performed in children did not discriminate age groups, our findings are uneasy to compare with other series. However, some characteristics of small children in our study were similar to what has been previously reported in Thailand [13] and Brazil [7]. In contrast, our results differ from a previous report of 43 children, 4–14 years of age [14] showing more severe manifestations of leptospirosis than the present study, including renal failure and thrombocytopenia. Several factors may explain these differences: first, different leptospiral serogoups were identified in New Caledonia; second, time to refer was potentially higher in the Brazilian study; finally, host factors and higher organism loads may lead to more severe manifestations. Conversely, presentation of severe illness in adolescents was similar to clinical and biological profile reported in adults in Brazil [3] and New Caledonia [8].

Consistent with some previous studies [5], [14], our results showed that overall case-fatality rates in children are lower than in adults. Recently identified factors associated with severe disease in adults in the same setting included tobacco use, leptospiral serogoup and delay between onset of symptoms and initiation of antibacterial therapy [8]. The discrepancy between children and adolescents does not appear related to differences in seroreactivity to leptospiral serogroups, which was similar in both groups. Similarly, onset of symptoms to administration of antibiotics was similar in both groups suggesting identical time to refer for children and adolescents. Host factors may contribute to this association and could include higher organism loads with increasing age. However, since risk factors for exposure were identical in children and adolescents, it is unlikely that initial bacterial inoculum was different in both groups. Age-dependant changes in innate and adaptative immune responses to leptospiral infection are plausible explanations for differences between children and adolescents. Several hypotheses regarding leptospirosis severity are based on host genetic susceptibility factors [15], [16] and/or on bacterial virulence [17], although the virulence mechanisms are poorly understood and are probably multifactorial [18].

The male predominance reported in our study both in children and adolescents is very similar to previous studies of symptomatic leptospirosis [3], [14]. Gender-specific activities may explain such differences. However, it is common in New Caledonia to see boys and girls swimming in water and assisting their parents with cultivating fields. The number of symptomatic cases admitted in paediatrics is low compared with adults over the same period of time [8]. This result is surprising since clinicians have a lower threshold to admit children with suspected or confirmed leptospirosis to the hospital. Age specific activities may explain this finding.

Another major finding of our study showed that JHRs were more likely to occur in adolescents. Although this adverse event is scarcely described in leptospirosis [19], our results support the presentation and outcome overlap between adolescents and adults. The incidence of JHR in children included in our study is in line with the few studies reporting JHR in paediatric cases [13]. In contrast, the incidence of JHR in adolescents was elevated when compared with other studies. JHR unobserved or unreported by clinicians are potential reasons for this reduced frequency. The delay before antibacterial therapy had a major impact on outcome in adults [8], [20]. The need for early initiation of antimicrobial therapy to reduce disease severity remains to be proven in children. However, presumptive treatment based on clinical and epidemiological evidence appears justified while waiting for the laboratory results. Modalities of antibiotics administration to prevent JHR to occur remain to be explored.

Our study suffers from several limitations. First, due to the retrospective design of the study, a significant proportion of patients with untraceable records were secondary excluded of the analysis. Second, leptospiral serogroups were identified in a limited number of cases only. Third, important clinical data were missing, including presence of puberty. Finally, indications for hospitalization may be different in younger children compared to adolescents and mild disease in adolescents may not have been seen because they were not hospitalized.

In New Caledonia, leptospirosis is responsible for a smaller number of hospitalizations in paediatrics due to milder symptomatic forms of the disease. However, leptospirosis remains a public health threat, including in the younger age group which can present with atypical signs and symptoms. Puberty may impact on the severity reported in the older age groups. Physiologic and immunologic factors associated with improved paediatric outcomes require further investigation to provide insights into the pathogenesis of severe leptospirosis.

## Supporting Information

Checklist S1 STROBE (checklist).
(DOC)
